# Clinical Outcomes in Scleral Fixation Secondary Intraocular Lens with Yamane versus Suture Techniques: A Systematic Review and Meta-Analysis

**DOI:** 10.3390/jcm13113071

**Published:** 2024-05-24

**Authors:** Charles Zhang, Charles Palka, Daniel Zhu, Daniel Lai, Jules Winokur, Treefa Shwani, Margaret M. DeAngelis, Andrew L. Reynolds

**Affiliations:** 1 Department of Ophthalmology, Ross Eye Institute, Jacobs School of Medicine and Biomedical Sciences, State University of New York, University at Buffalo, Buffalo, NY 14203, USA; czhang62@buffalo.edu (C.Z.); dalai2@buffalo.edu (D.L.); treefash@buffalo.edu (T.S.); alr23@buffalo.edu (A.L.R.); 2 Jacobs School of Medicine and Biomedical Sciences, State University of New York at Buffalo, 955 Main Street, Buffalo, NY 14203, USA; cpalka@buffalo.edu; 3 Department of Ophthalmology, Northwell Health Eye Institute, Great Neck, NY 11021, USA; dzhu@northwell.edu (D.Z.); jwinokur@northwell.edu (J.W.); 4 Neuroscience Graduate Program, Jacobs School of Medicine and Biomedical Sciences, State University of New York, University at Buffalo, Buffalo, NY 14203, USA; 5 Department of Ophthalmology and Visual Sciences, University of Utah School of Medicine, The University of Utah, Salt Lake City, UT 84132, USA; 6 Department of Population Health Sciences, University of Utah School of Medicine, The University of Utah, Salt Lake City, UT 84132, USA; 7 Veterans Administration Western New York Healthcare System, Buffalo, NY 14212, USA; 8 Department of Biochemistry, Jacobs School of Medicine and Biomedical Sciences, State University of New York, University at Buffalo, Buffalo, NY 14203, USA; 9 Genetics, Genomics and Bioinformatics Graduate Program, Jacobs School of Medicine and Biomedical Sciences, State University of New York, University at Buffalo, Buffalo, NY 14203, USA

**Keywords:** secondary IOL, Yamane, scleral fixation, sutureless fixation, flanged haptic

## Abstract

**Background:** The purpose of the study is to compare the visual outcomes and complications of sutured scleral fixation (SSF), a traditional and conservative surgical approach, and the newer and faster Yamane technique for secondary intraocular lens placement. **Methods:** A literature search was performed on PubMed, Embase, and Scopus on studies published between 1 July 2017 to 29 September 2023. Outcomes analyzed included the final best corrected visual acuity (BCVA) between 3 and 12 months to assess the effectiveness of the procedure, post-operative month (POM) 1 BCVA to assess the speed of visual recovery, endothelial cell count (ECC), absolute refractive error, surgical duration, and complication rates. Additional subgroup analyses were performed based on surgeon experience with the technique. Single-surgeon studies had an average of 26 procedures performed, whereas multiple-surgeon studies averaged only 9 procedures performed; these were then used to delineate surgeon experience. A sample-size weighted mean difference (MD) meta-analysis was performed across all variables using RevMan 5.4.1; *p* < 0.05 was considered statistically significant. **Results:** Thirteen studies with 737 eyes were included: 406 eyes were included in the SSF group, and 331 eyes were included in the Yamane group. There was no significant difference in the final BCVA between groups in both the single-surgeon versus multiple-surgeon studies (MD = −0.01, 95% CI: [−0.06, 0.04], *p* = 0.73). In the single-surgeon studies, the BCVA at POM1 was significantly improved in the Yamane group compared to SSF (MD = −0.10, 95% CI: [−0.16, −0.04], *p* = 0.002). In the multiple-surgeon studies, there was no significant difference in BCVA at POM1 (MD = −0.06, 95% CI: [−0.16, 0.04], *p* = 0.23). The Yamane group had a shorter surgical duration than SSF in both single-surgeon and multiple-surgeon studies (MD = −24.68, 95% CI: [−35.90, −13.46], *p* < 0.0001). The ECC, refractive error, and complication rates did not significantly differ amongst all groups. **Conclusions:** The Yamane technique demonstrated similar long-term visual outcomes and complication rates to the traditional SSF. Visual recovery was significantly faster in the Yamane group in the single-surgeon studies. The operative times were shorter across all Yamane groups. Based on these findings, it is advisable to consider the Yamane technique as a viable, and perhaps preferable, option for patients requiring secondary IOL placement, alongside traditional SSF methods.

## 1. Introduction

Secondary intraocular lens (IOL) placement is needed in conditions of posterior capsule inadequacy or zonular weakness [1]. Such conditions can arise from trauma; inherited conditions such as Marfan’s syndrome, pseudoexfoliation syndrome, or congenital aniridia; as well as complications from cataract surgery [2]. As the global population grows older, the frequency of cataract procedures is increasing, leading to a rise in the rate of secondary IOL implantation [3]. Secondary IOL implantation after cataract surgery may be required due to intraoperative damage to the zonules or lens capsule or post-operative IOL dislocation in patients with pseudoexfoliation syndrome or trauma [1,4,5].

Over the last 70 years, a range of secondary IOL techniques have been developed. These include anterior chamber IOL (ACIOL), iris-fixated IOL (IFIOL), sutured scleral fixation (SSF) IOL, and sutureless scleral fixated IOL, each with their own set of benefits and risks [1,6]. ACIOLs are often viewed as the most technically straightforward approach but can lead to corneal endothelial cell decompensation over time [6,7,8,9]. Furthermore, if not sized correctly, they can lead to uveitis–hyphema–glaucoma syndrome [9]. IFIOLs are considered more technically challenging than ACIOLs but have been associated with the increased risk of an anatomic distortion of the pupil, uveitis from the pigment release, as well as vitreous hemorrhage [1,5,10]. Scleral-fixated IOLs are considered the most technically challenging technique and offer a significantly lower risk of damage to the anterior segment structures [11]; however, suture-related complications, such as suture erosion and breakage, can occur, which can potentially lead to the dislocation of the IOL [11,12,13,14].

A notable advancement in the field is the development of sutureless scleral fixation. Introduced by Maggi and Maggi in 1997, and most recently refined by Yamane et al. in 2017, this technique employs the creation of parallel scleral tunnels using 29G or 30G thin-walled needles [15,16,17]. Instead of sutures, the haptics of a three-piece IOL are externalized through these tunnels and intrasclerally fixated through the use of flanges created via cauterization at the ends of the haptics [17]. However, this technique is not without risks as many surgeons have reported on complications such as cystoid macular edema, corneal edema, increased IOP, vitreous hemorrhage, lens tilt, and haptic erosion leading to dislocation [18,19]. Given these dynamics, there is a clear need to compare the efficacy and risks associated with traditional SSF and the Yamane technique.

Since the publication of the Yamane technique, numerous studies have been published comparing the two procedures in terms of outcomes and complications [5,20,21,22,23,24,25,26,27,28,29,30,31]. Studies have demonstrated that surgeons with greater experience in specific ophthalmic techniques have more favorable results with improved visual recovery after surgery, and, thus, it is important to consider surgeon experience when comparing these two methods [32,33]. This meta-analysis aims to rigorously evaluate and compare the Yamane technique against traditional SSF across various parameters including visual acuity, refractive outcome, procedure time, and complication rates.

## 2. Materials and Methods

### 2.1. Design

We performed our search in line with the Preferred Reporting Items for the Systematic Reviews and Meta-Analyses (PRISMA) statement guidelines and Cochrane Handbook of Interventions chapter on screening [34,35]. We prospectively submitted our study protocol to an online database for systematic reviews, the international prospective register of systematic reviews (PROSPERO ID: CRD42023466373). Ethical approval was not sought because these meta-analyses did not access private information. Informed consent was not sought because the present review analyzed already published studies that had obtained informed consent for their respective participants.

### 2.2. Search Strategy

PubMed, Embase, and Scopus were searched between 27–29 September 2023. All three databases were searched via the web. Queries included the terms “Yamane”, “suture-less”, “scleral sutured”, “secondary intraocular lens”, and “flanged fixation” for articles published since 1 July 2017, when the Yamane technique was first described. The full search strategies can be found in Supplemental Table S1.

### 2.3. Article Selection

In the first phase of screening, two authors (CP and DL) independently screened titles and abstracts of the results from our searches on the three databases. During this phase of screening, articles were selected if they directly compared the Yamane to SSF in the title or abstract.

In the second phase, the two authors screened full texts independently using the following predetermined inclusion and exclusion criteria.

The inclusion criteria are as follows: (1) Adult patients (>18 years old); (2) patients receiving secondary intraocular lens implantation by either Yamane flanged intrascleral fixation or by scleral suture fixation; (3) sample sizes of at least 10 patients; (4) patients with post-operative record of best corrected visual acuity; (5) prospective and retrospective comparative studies; and (6) no language restrictions.

The exclusion criteria are as follows: (1) duplicate literature or data—if multiple articles review the same data, the most recent publication was used, (2) non-human studies; (3) meta-analyses, review articles, and case reports; and (4) non-published data (abstracts, presentations, and conference proceedings).

Disagreements between the two primary investigators are settled by third party overseeing the meta-analysis (CZ/DZ). This occurred in only one study which was ultimately excluded.

**Figure 1:**
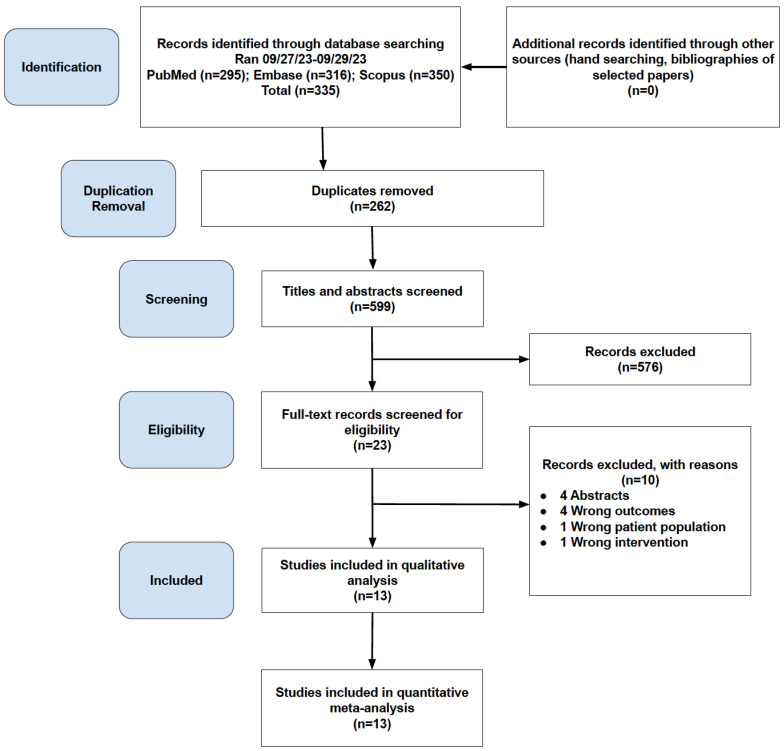
Preferred Reporting Items for Systematic Reviews and Meta-Analyses (PRISMA) flowchart.

The database searches of PubMed, Embase, and Scopus yielded 295, 316, and 350 results, respectively. After removal of duplicates, the final number of studies included for screening of titles and abstracts was 599. A total of 262 duplicates were removed during importation. After title and abstract screening, the total number of studies was 23. Full-text review narrowed this number down to 13 studies that fit our inclusion and exclusion criteria. Data were extracted from these 13 studies. The PRISMA flowchart in Figure 1 illustrates the above process of importation, screening, and data extraction of the studies included in the meta-analysis.

### 2.4. Quality Assessment

The Risk of Bias in Non-Randomized Studies—of Interventions (ROBINS-I) tool was used to assess risk of bias in retrospective and prospective non-randomized studies. The NIH Quality Assessment Tool of Controlled Intervention Studies was used for prospective randomized studies. These tools were used by two authors independently (CP and DL) [36,37,38,39,40].

### 2.5. Data Extraction

Three authors (CP, CZ, and DZ) analyzed the 13 studies for data extraction. Conflicts were resolved through consensus. Data points collected include name of study, authors, year of publication, country, sample size, best corrected visual acuity (BCVA), endothelial cell count (ECC), surgical duration, secondary surgeries, and complication rates. To account for surgeon experience, we stratified the studies by number of surgeons if they explicitly stated the number of surgeons, or using the initials next to each procedure. Elsayed et al. did not report the number of surgeons or initials of surgeons, and we were unable to obtain a response when we reached out to the authors. Therefore, it was not included in either the single- or multiple-surgeon subgroups. Microsoft Excel v16.18 was used to compile all extracted data.

The POM1 visit was also extracted in addition to the final BCVA. Studies have demonstrated that most SSFs require multiple months for complete visual recovery [5,20,22,23,24,25,29,30]. As such, earlier post-operative visits can be used as a method to assess visual recovery. Most intraocular lens studies often report a POM1 visit as it is an established standard of care across ophthalmic surgery and, therefore, most likely to be consistently reported across studies [41,42].

### 2.6. Statistical Analysis

A sample-size weighted mean difference (MD) meta-analysis was performed for comparing final BCVAs, POM1 BCVA, post-operative ECC, and absolute SE error, as well as mean surgical durations between the Yamane and SSF treatment groups using the RevMan 5.4.1 software (Cochrane Collaboration, Copenhagen, Denmark) as previously described [7,43]. The RevMan 5.4.1 software was also used for meta-analysis of risk ratios (RRs) to compare complication rates, including CME and secondary surgery rates between Yamane and SSF techniques as previously described [11,43,44]. Due to the anticipated heterogeneity secondary to the variation in the protocols, follow-up durations, IOL types, and surgical technique, the random-effects model (DerSimonian and Laird method) was utilized. Forest plots were used to depict the summary effect measure and 95% CI. Leave-one-out sensitivity analyses were conducted by removing a single study at a time involved in each meta-analysis and examining the corresponding pooled WMDs or RRs in order to determine its impact. Heterogeneity was determined using Cochran’s Q and Higgins’ I^2^. Significant heterogeneity was defined as a Cochran’s Q *p* value of <0.1 and an I^2^ > 40%. In cases where heterogeneity was detected, subgroup analysis was performed [45,46]. A *p* value < 0.05 was considered statistically significant and all statistical analyses were two-sided. One study reported their results using median and range [30]. To estimate the means and standard deviations of this study, a previously described formula was used [47,48,49].

### 2.7. Publication Bias

Using RStudio (Version 2023.03.01 Build 446), a Begg’s funnel plot and Egger’s regression analysis were conducted to assess for asymmetry and publication bias.

## 3. Results

### 3.1. Study Characteristics

The thirteen studies used for the present meta-analysis were published from 2021 to 2023 and included 737 eyes (331 Yamane, 406 SSF). Ten of the thirteen studies were retrospective cohort analyses [20,22,23,24,25,26,27,28,30,31], while three were prospective [5,21,29]. One of the prospective studies was randomized. The studies were conducted in the United States (1), South Korea (7), India (1), China (1), Egypt (1), Germany (1), and Turkey (1). The studies reported on BCVA, ECC, refractive outcomes, surgical durations, and complication rates. A summary of the included studies is depicted in Table 1.

### 3.2. ROBINS-I, NIH Quality Assessment, and GRADE Assessment

The Risk of Bias in Non-Randomized Studies—of Interventions (ROBINS-I) tool was used to assess the risk of bias of both retrospective and prospective non-randomized studies. The NIH Quality Assessment of Controlled Intervention Studies tool was used for the prospective study. Disputes between reviewers were settled with a third and final review of the disputed studies. After an analysis of the retrospective studies with the ROBINS-I tool, a moderate risk of bias was assessed for the following domains: confounding variables, selection of participants, measurement of outcomes, and results reporting. The classification of intervention, deviation from intervention, and missing data were determined to be low-risk. The results of this analysis are shown in Table S2.

After an analysis of the prospective studies with the NIH Controlled Interventions tool, the following domains were found to be moderate-risk: the randomization of interventions, technique of randomization, treatment allocation, blinding of providers/participants, blinding of outcomes assessment, and sufficient sample size to detect a difference with at least 80% power. The baseline characteristics of study participants, overall and differential dropout rates, intervention adherence, background treatments, consistent/valid/reliable measures of outcome, pre-specified outcomes and subgroup analysis, and intention-to-treat domains were all considered low-risk. The results of this analysis shown in Table S3. The certainty of the evidence for the outcomes was assessed using the Grading of Recommendations, Assessment, Development and Evaluations (GRADE) framework similar to the previously described work, and determined to be very low to low, as depicted in Table S4 [50].

### 3.3. Final Visual Outcomes of Yamane versus Sutured Scleral Fixation

**Figure 2:**
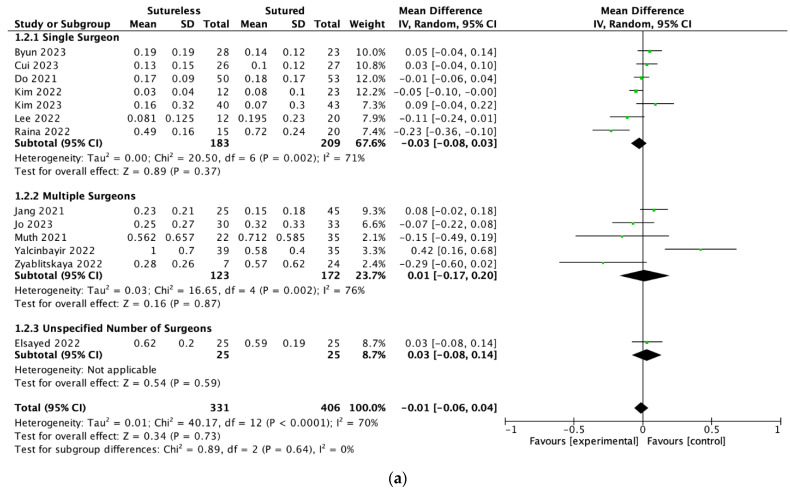

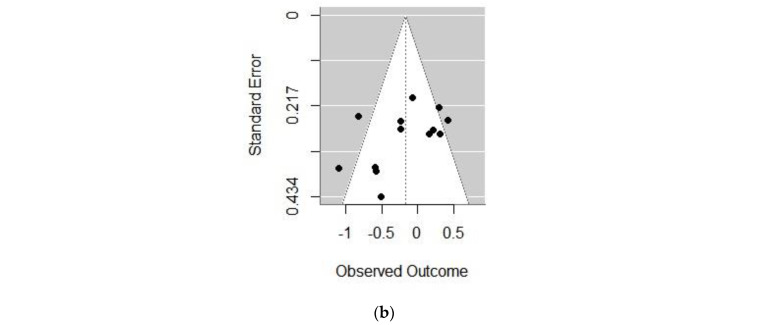
(**a**) Forest plot meta-analysis comparing the best corrected visual acuities (BCVAs) at last follow-up between Yamane versus sutured scleral fixation. Each study is shown by the last name of the first author and the mean difference (MD) with 95% confidence interval (CI) [5,20,21,22,23,24,25,26,27,28,29,30,31]. The summary mean difference and 95% CI are also shown (according to random-effect estimations). For subgroup analysis, studies are separated by single surgeon, multiple surgeons, or unspecified number of surgeons. (**b**) Funnel plot of the studies included in the meta-analysis. IV = inverse variance; SD = standard deviation.

A meta-analysis of the data extracted from 13 studies comparing the final recorded BCVA showed that there was no significant difference between Yamane and SSF (MD = −0.01, 95% CI: [−0.06, 0.04], *p* = 0.73). There was significant heterogeneity detected (I^2^ = 70%, *p* < 0.0001). Because of this heterogeneity, we conducted a subgroup analysis based on the number of surgeons involved in each study. When these groups were stratified based on the number of surgeons, there was no significant difference in the final visual acuity between treatment groups in the single-surgeon study and the multiple-surgeon studies (Single surgeon: MD = −0.03, 95% CI: [−0.08, 0.03], *p* = 0.37; Multiple surgeons: MD = 0.01, 95% CI: [−0.17, 0.20], *p* = 0.87). Between subgroups, there was no significant heterogeneity detected (I^2^ = 0%, *p* = 0.64). The forest plot with a subgroup analysis is shown in Figure 2a. The number of Yamane and SSF cases per surgeon in the single-surgeon group were 26.1 and 29.9, respectively. The number of Yamane and SSF cases per surgeon in the multiple-surgeon group were 9.5 and 13.2, respectively. The pooled MD remained statistically insignificant with each study removed in the sensitivity analysis. A linear Egger regression analysis of funnel plot asymmetry did not detect any publication bias in the final BCVA results (*p* = 0.138, Figure 2b).

### 3.4. Visual Outcomes of Yamane versus Sutured Scleral Fixation at 1 Month

**Figure 3:**
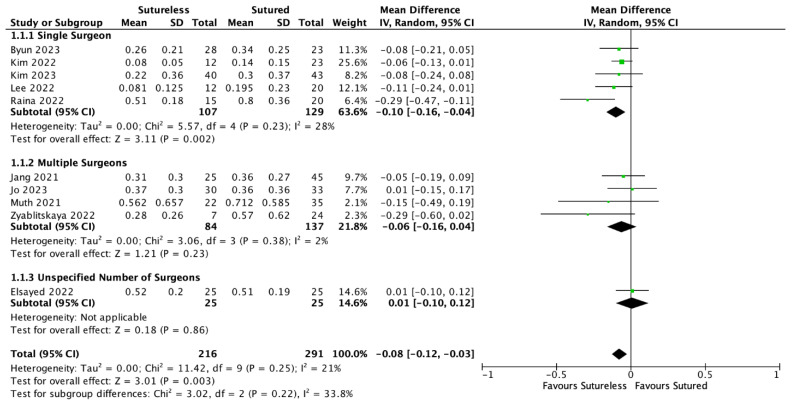
Forest plot meta-analysis comparing the best corrected visual acuities (BCVAs) at 1 month between Yamane versus sutured scleral fixation. Each study is shown by the last name of the first author and the mean difference (MD) with 95% confidence interval (CI) [20,22,23,24,25,26,27,28,29,31]. The summary mean difference and 95% CI are also shown (according to random-effect estimations). For subgroup analysis, studies are separated by single surgeon, multiple surgeons, or unspecified number of surgeons. IV = inverse variance; SD = standard deviation.

A meta-analysis of the data extracted from 10 studies comparing the BCVA at 1 month showed that Yamane had significantly better outcomes than SSF, with an average difference of 0.08 LogMAR (MD = −0.08, 95% CI: [−0.12, −0.03], *p* = 0.003). In multiple-surgeon studies, there was no significant difference between the two techniques (MD = −0.06, 95% CI: [−0.16, 0.04], *p* = 0.23). There was a significant difference between the BCVA at 1 month in the single-surgeon group (MD = −0.10, 95% CI: [−0.16, −0.04], *p* = 0.002). The forest plot with a subgroup analysis is shown in Figure 3. The pooled MD remained statistically significant with each study removed in the sensitivity analysis. There was no significant heterogeneity detected (I^2^ = 21%, *p* = 0.25). 

### 3.5. Refractive Outcomes of Yamane versus Sutured Scleral Fixation

**Figure 4:**
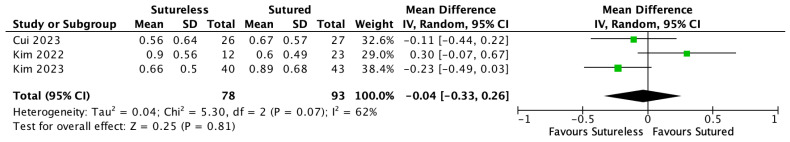
Forest plot meta-analysis comparing the absolute difference between the intended and final spherical equivalent between Yamane versus sutured scleral fixation. Each study is shown by the last name of the first author and the mean difference (MD) with 95% confidence interval (CI) [21,25,26] The summary mean difference and 95% CI are also shown (according to random-effect estimations). IV = inverse variance; SD = standard deviation.

The absolute refractive error was defined as the absolute difference between the intended spherical equivalent and final spherical equivalent. A meta-analysis of the data identified three studies that reported the absolute refractive error. There was no significant difference between the absolute refractive errors between Yamane and SSF (MD = −0.04, 95% CI: [−0.33, 0.26], *p* = 0.81). The pooled MD remained statistically insignificant with each study removed in the sensitivity analysis. Heterogeneity was detected in the analysis of the absolute refractive error (I^2^ = 62%, *p* = 0.07). The forest plot is shown in Figure 4. There was no subgroup analysis carried out for this group as there were only three studies reporting the absolute refractive error.

### 3.6. Surgical Duration of Yamane versus Sutured Scleral Fixation

**Figure 5:**
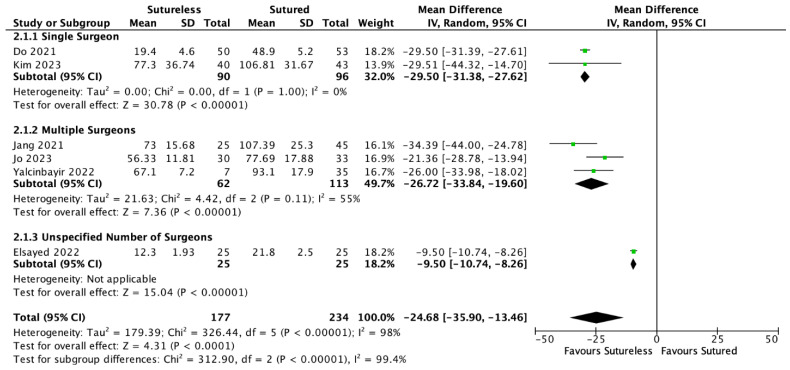
Forest plot meta-analysis comparing the mean surgical durations between Yamane versus sutured scleral fixation. Each study is shown by the last name of the first author and the mean difference (MD) with 95% confidence interval (CI) [5,22,23,24,26]. The summary mean difference and 95% CI are also shown (according to random-effect estimations). For subgroup analysis, studies are separated by single surgeon, multiple surgeons, or unspecified number of surgeons. IV = inverse variance; SD = standard deviation.

A meta-analysis of data extracted from six studies found that the mean surgical time was significantly shorter in the Yamane group than the SSF group by an average of 24.68 min. (MD = −24.68, 95% CI: [−35.90, −13.46], *p* < 0.00001). There was significant heterogeneity (I^2^ = 98%, *p* < 0.0001), so a subgroup analysis was conducted based on the number of surgeons included in the studies. In both the single- and multiple-surgeon subgroups, the Yamane group’s surgical duration was significantly shorter than SSF (Single surgeon: MD = −29.50, 95% CI: [−31.38, −27.62], *p* < 0.00001; Multiple surgeon: MD = −26.72, 95% CI: [−33.84, −19.60], *p* < 0.00001). The pooled MD remained statistically significant with each study removed in the sensitivity analysis. The forest plot with a subgroup analysis is shown in Figure 5. Mean Yamane durations ranged from 12.3 to 77.3 min. Mean SSF durations ranged from 21.8 to 107.39 min. The wide range of durations is due to some studies including pars plana vitrectomy in their surgical duration calculation and others not including it. The studies’ authors were consistent with reporting surgical duration in that either all cases included PPV or anterior vitrectomy in their calculation of duration or not. Therefore, the difference in surgical duration can be attributed to the difference between fixation techniques rather than the time spent on vitrectomies. We excluded Muth et al. from the surgical duration calculations because it was unclear if all patients were aphakic or underwent PPV prior to secondary IOL implantation [28]. The only source of variability in duration in these groups would come from the difference between Yamane and SSF.

### 3.7. Endothelial Cell Count

**Figure 6:**
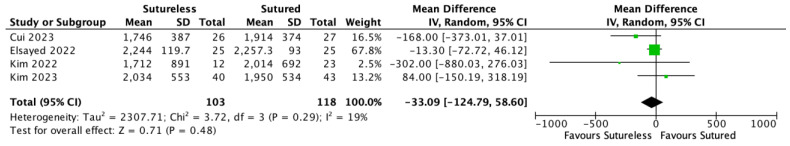
Forest plot meta-analysis comparing the post-operative endothelial cell count (ECC) between Yamane versus sutured scleral fixation. Each study is shown by the last name of the first author and the mean difference (MD) with 95% confidence interval (CI) [21,22,25,26]. The summary mean difference and 95% CI are also shown (according to random-effect estimations). IV = inverse variance; SD = standard deviation.

A meta-analysis of data extracted from four studies found there to be no significant difference between post-operative ECC between the Yamane and SSF techniques (MD = −33.09, 95% CI: [−124.79, 58.60], *p* = 0.48). The forest plot is shown in Figure 6. The pooled MD remained statistically insignificant with each study removed in the sensitivity analysis. No significant heterogeneity was detected (I^2^ = 19%, *p* = 0.29).

### 3.8. Complications of Yamane Technique

**Figure 7:**
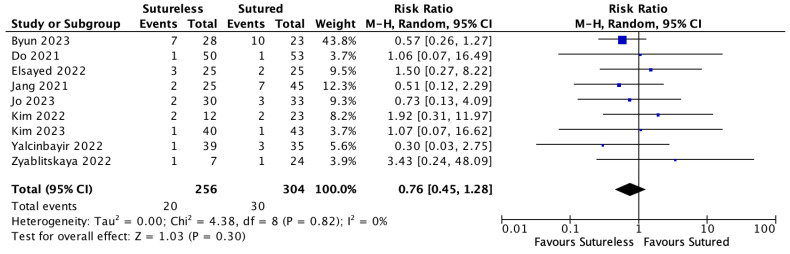
Forest plot of the risk ratio (RR) meta-analysis comparing rates of cystoid macular edema (CME) between Yamane versus sutured scleral fixation. Each study is shown by the last name of the first author and the RR with 95% confidence interval (CI) [5,20,22,23,24,25,26,30,31]. The combined effect and 95% CI are also shown (according to random-effect estimations). IV = inverse variance.

**Figure 8:**
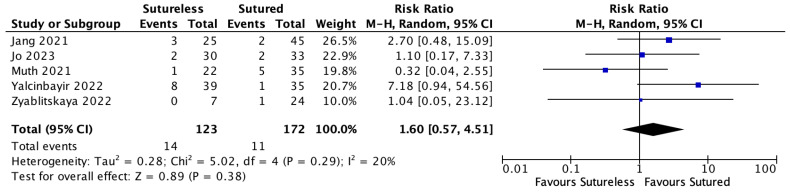
Forest plot of the risk ratio (RR) meta-analysis comparing rates of secondary surgical intervention between Yamane versus sutured scleral fixation. Each study is shown by the last name of the first author and the RR with 95% confidence interval (CI) [23,24,28,30,31]. The combined effect and 95% CI are also shown (according to random-effect estimations). IV = inverse variance.

There is no significant difference between the rates of cystoid macular edema (RR: 0.76 [0.45, 1.28], *p* = 0.30) between the two groups. There was no significant heterogeneity detected (I^2^ = 0%, *p* = 0.82). The forest plot of cystoid macular edema risk ratios is shown in Figure 7. There was also no significant difference between the rates of secondary surgical intervention (RR: 1.60 [0.57, 4.51], *p* = 0.38). The forest plot of secondary surgical intervention risk ratios is shown in Figure 8. The pooled RRs remained statistically insignificant with each study removed in the sensitivity analysis. There was no significant heterogeneity detected for secondary surgical interventions (I^2^ = 20%, *p* = 0.29). There were three post-operative cases of retinal detachment (RD) (1 in Yamane, 2 in SSF). Due to the paucity of RD and vitreous hemorrhage events, with the majority of studies reporting zero events in both treatment groups, an RR meta-analysis was unable to be performed. A summary of the cumulative complications across studies is depicted in Table 2.

## 4. Discussion

In this meta-analysis, the Yamane technique resulted in a similar final BCVA to traditional SSF. The final BCVA in the traditional SSF was also consistent with other meta-analyses on scleral-fixated IOLs in the literature, supporting the robustness of our analysis [51]. Other meta-analyses have established that alternative secondary IOL techniques such as ACIOL and IFIOL can result in visual outcomes comparable to the gold-standard of SSF; however, they report greater rates of complications [11,51]. In our meta-analysis, we report no significant differences in complications, including dislocation rates, retinal detachment, CME, or vitreous hemorrhage, nor any difference in refractive outcomes, averaging within 1.00 D of emmetropia. Notably, we found the Yamane technique consistently demonstrated a shorter operative time across all studies and faster visual recovery at POM1 in experienced surgeons.

In our meta-analysis, we performed subgroup analysis based on the number of surgeons when evaluating the visual recovery at POM1 BCVA in patients, the rationale being that the single surgeon performed, on average, 26 Yamane procedures, whereas the multiple-surgeon group averaged only 9 Yamane procedures per surgeon. The single-surgeon groups displayed superior POM1 BCVA as compared to the SSF group; however, when it came to the final BCVA, both the single-surgeon and multiple-surgeon Yamane groups were not significantly different compared to SSF. We believe that the difference in the single-surgeon Yamane group at POM1 may be due to the reduced corneal edema as prior studies have reported on the effect of surgeon experience on limiting post-operative corneal edema [32]. The extensive manipulation of the globe, such as with scleral-fixated IOL techniques, as well as protracted anterior segment surgical durations, can lead to increased inflammation and corneal edema in the early post-operative period [52,53]. We were able to demonstrate that the Yamane technique had significantly shorter operative times which may be contributing to the observed difference at POM1; however, the surgeon inexperience in the multiple-surgeon group may have resulted in unnecessary globe manipulation or longer operative times that could not be captured in our study [52,53]. Interestingly, we did not observe a significant disparity in ECC loss between groups.

A significant concern of sutureless scleral fixation techniques relates to potential IOL tilt, decentration, and dislocation [17,54]. Although the first two issues can be addressed with refractive lenses, the last issue often requires additional surgery. The Yamane technique attempts to mitigate these challenges through the use of angled scleral tunnels. Stem et al. performed a study on cadaveric eyes to examine forces required for the disinsertion of flanged versus un-flanged haptics. They found that flanged haptics require significantly more force for disinsertion and, ultimately, dislocation [55]. Despite this, there remains uncertainty on the long-term stability of Yamane fixation [29]. In the studies comparing Yamane to SSF, the IOL tilt was measured via ultrasound bio-microscopy or AS-OCT, while IOL centration was measured via a slit lamp exam [20,21,26,27,28,29,31]. Cui et al. used AS-OCT to quantitatively measure IOL tilt and found it to be 8.2 degrees in the Yamane group and 8.22 degrees in the SSF group. They also found that there was a decentration of 0.36 mm in both the Yamane and SSF groups in the horizontal direction over 12 months [21]. This variable was also indirectly evaluated by examining the final refractive error, as the implanted lens instability could influence the final refraction [56]. Although only three studies reported the absolute refractive error relative to their target refraction, we found no significant difference between the Yamane and SSF techniques [21,25,26]. While data in this domain are somewhat limited, our study’s overarching implication is that there is no discernible increase in the rates of IOL dislocation or decentration with the Yamane technique when compared with SSF.

In addition to surgery for IOL dislocation, secondary IOLs can occasionally lead to corneal decompensation or vitreous hemorrhage, necessitating additional surgery [1]. In our study, the SSF and Yamane techniques exhibited no significant disparities in terms of the need for secondary surgery. Specifically, the Yamane group required 14 additional surgical procedures compared to 11 in the SSF group, which represented an overall rate of 4.2% and 2.7%, respectively. Only two studies reported higher rates of secondary surgical intervention in the Yamane group [23,30]. One such study was that of Yalcinbayir et al., who reported a total of eight eyes requiring additional surgical intervention in the Yamane group. They mentioned that a majority of these occurred in the first 15 cases performed by their surgeons [30]. Four of these surgeries involved the repositioning of the three-piece IOL [30]. This pattern suggests a steep learning curve for the Yamane technique. This might indicate that experience with the technique is necessary in order to minimize IOL dislocation and the requirement of secondary surgery.

A unique concern of the Yamane technique is haptic extrusion from the scleral tunnels which typically requires additional surgery with an explanation of the IOL. Lin et al. observed haptic extrusion in three instances (16%) within a 24-month follow-up period [57]. Interestingly, all three cases were identified in profoundly myopic eyes, suggesting possibly avoiding the Yamane technique for such patients or other patients with thinned sclera. Within the scope of our meta-analysis, no instances of haptic extrusion were reported. However, this absence might be attributable to the relatively brief follow-up durations, which were, at most, 12 months.

There was a significant amount of heterogeneity in some of the outcomes, especially mean surgical duration. This was due to some of the studies including pars plana vitrectomy in the surgical duration calculation. Other studies involved patients who were aphakic, thus leading to a shorter surgery time. Moreover, lens recovery from a prior dislocation or subluxation was included in some calculations and may have increased mean surgical times. Despite this heterogeneity between studies, the Yamane technique was considerably faster than SSF in all the studies we evaluated.

This meta-analysis has several limitations related to the design of the selected studies and sample sizes. The first limitation was that ten of the studies were retrospective cohort analyses. The possible disadvantages of this are the lack of ability to control for parameters like follow-up duration or the surgeon performing the secondary IOL fixation. There were only three prospective studies. Only one of these prospective studies was randomized. With a predominance of retrospective and non-randomized prospective studies, there was a concern for reporting and selection bias. More prospective, randomized studies will be necessary to control for these biases. The choice of surgical technique was also left up to the surgeon in both the retrospective and prospective analyses. This could lead to a significant selection bias in that patients with pre-operative risk factors may have been preferentially grouped into one treatment category. Another limitation of this analysis is the relatively small sample sizes across most included studies. A meta-analysis with larger sample sizes would provide more convincing insight into the complication rates of these techniques. Another limitation of this analysis is the lack of uniformity amongst the surgeries. Because it is up to the surgeon’s preference, nearly every study used a different combination of lenses, sutures, and gauges to perform the sclerotomies. The Yamane technique was more uniform than the SSF technique with most surgeons using a Sensar AR40e lens and a 29G or 30G thin-walled needle. The methods for reporting visual acuity, absolute refractive error, and mean surgical duration also differed amongst studies. For example, researchers from one study might report pre-operative BCVA, and then BCVA at 1 month and 6 months, whereas another study might report only the pre-operative BCVA and BCVA at 12 months. Future studies are necessary in order to investigate longer-term outcomes. The longest follow-up period in the meta-analysis was 12 months. As these lenses are meant to provide vision for periods longer than 12 months, it would be valuable to understand the visual and refractive outcomes in a multi-year study.

To conclude, the present meta-analysis demonstrated comparable final visual outcomes and complication rates between the Yamane and traditional SSF. Yamane was shown to have shorter surgical durations and better visual recovery in the hands of experienced surgeons. Based on these findings, it is advisable to consider the Yamane technique as a viable, and perhaps preferable, option for patients requiring secondary IOL placement, alongside traditional SSF methods.

## Figures and Tables

**Table 1:** Summary of Included Studies.

Source (Author Year)	Study Design	Techniques	Country	Mean Age	Number of Eyes	Last Follow-Up (Months)
Byun 2023 [20]	Retrospective	Trocar-cannula-based sutureless, sutured scleral fixated	South Korea	61.37 ± 10.95; 60.86 ± 11.45 (sutureless), 62.00 ± 10.54 (sutured)	58 (28 sutureless, 23 sutured)	12
Cui 2023 [21]	Prospective, non-randomized	Yamane, sutured scleral fixation	China	56.5 ± 13.9 (sutureless), 56.5 ± 10.1 (sutured)	53 (26 sutureless, 27 sutured)	12
Do 2021 [5]	Prospective, randomized	Yamane, sutured scleral fixation	South Korea	63.3 ± 7.0 (sutureless), 64.1 ± 6.9 (sutured)	103 (50 sutureless, 53 sutured)	12
Elsayed 2022 [22]	Retrospective	Flanged haptic fixation, sutured scleral fixated	Egypt	61.1 ± 6.41 (sutureless), 59.2 ± 8.20 (sutured)	50 (25 sutureless, 25 sutured)	6
Jang 2021 [23]	Retrospective	Yamane, sutured scleral fixation	South Korea	61.49 ± 11.89; 62.92 ± 9.91 (sutureless), 60.68 ± 12.92 (sutured)	70 (25 sutureless, 45 sutured)	6
Jo 2023 [24]	Retrospective	Yamane, sutured scleral fixation	South Korea	61.23 ± 9.22 (sutureless), 61.82 ± 8.20 (sutured)	69 (30 sutureless, 33 sutured)	6
Kim 2022 [25]	Retrospective	Yamane, sutured scleral fixation	South Korea	61.2 ± 8.7 (sutureless), 56.8 ± 11.6 (sutured)	35 (12 sutureless, 23 sutured)	6
Kim 2023 [26]	Retrospective	Yamane, sutured scleral fixation (10-0 polypropylene)	South Korea	60.75 ± 10.71 (sutureless) 59.16 ± 12.50 (sutured)	83 (40 sutureless, 43 sutured)	3
Lee 2022 [27]	Retrospective	Yamane, sutured scleral fixation	South Korea	65.920 ± 13.942 (sutureless), 58.350 ± 12.787 (sutured)	32 (12 sutureless, 20 sutured)	1
Muth 2021 [28]	Retrospective	Prolene suture, Gore-Tex suture, Yamane	Germany	63.8 ± 19.4; 68.4 ± 15.4 (sutureless), 62.8 ± 22.4 (Prolene), 58.7 ± 20.4 (Gore-Tex)	57 (22 sutureless, 20 Prolene, 15 Gore-Tex)	N/A
Raina 2022 [29]	Prospective, randomized	Yamane, sutured scleral fixation (Gore-Tex)	India	46.67 ± 16.97 (sutureless), 27.15 ± 13.29 (sutured)	35 (15 sutureless, 20 sutured)	6
Yalcinbayir 2022 [30]	Retrospective	Yamane, sutured scleral fixation	Turkey	61.6 ± 19.2 (sutureless), 53.9 ± 19.2 (sutured)	74 (39 sutureless, 35 sutured)	12
Zyablitskaya 2022 [31]	Retrospective	Two-point sutureless (CTL IOL), four-point sutured (AK IOL)	United States	68 ± 19.62	31 (7 sutureless, 24 sutured)	1

**Table 2:** Summary of complications.

Yamane Technique Complications		Scleral-Sutured Complications	
Cystoid Macular Edema	20	Cystoid Macular Edema	30
Increased IOP	8	Increased IOP	16
Vitreous Hemorrhage	7	Vitreous Hemorrhage	9
Suture/Haptic Complications	5	Suture/Haptic Complications	8
Retinal Detachment	1	Retinal Detachment	2
Glaucoma	1	Glaucoma	1
Corneal Edema	4	Corneal Edema	1
Hyphema	2	Hyphema	0
IOL Decentration	5	IOL Decentration	0

## Data Availability

All data contained within the article.

## Supplementary Materials

The following supporting information can be downloaded at: https://www.mdpi.com/article/10.3390/jcm13113071/s1, Table S1. Search Strategies. Table S2. The Risk of Bias in Non-Randomized Studies-of Interventions (ROBINS-I) tool. Table S3. NIH Quality Assessment of Controlled Intervention Studies. Table S4. GRADE Certainty Assessment and Summary of Findings.
