# Supplementary material for: Clinical Outcomes in Scleral Fixation Secondary Intraocular Lens with Yamane versus Suture Techniques: A Systematic Review and Meta-Analysis
Source: J Clin Med. 2024 May 24;13(11):3071. doi: 10.3390/jcm13113071 (PMC11173341; doi:10.3390/jcm13113071)
Supplement: Supplementary file 1 [file jcm-13-03071-s001.zip › jcm-2819610-supplementary.pdf]

Table S1. Search Strategies.

Database	Query	Date	Results
Pubmed	(Yamane OR flanged OR (suture-less) OR sutureless) AND ((scleral fixat*) OR (secondary intraocular lens) OR (secondary IOL)) OR (flanged fixation)	27-Sep	295
Embase	('yamane' AND 'scleral-sutured' OR 'sutureless' AND 'sutured') OR 'secondary IOL'	27-Sep	316
Scopus	(TITLE-ABS-KEY (yamane) OR TITLE-ABS-KEY (flanged) OR TITLE-ABS-KEY (sutureless) OR TITLE-ABS-KEY (suture-less)) AND (TITLE-ABS-KEY (scleral AND fixat*) OR TITLE-ABS-KEY (secondary AND iol) OR TITLE-ABS-KEY (secondary AND intraocular AND lens)) AND PUBYEAR > 2016 AND PUBYEAR < 2024	27-Sep	250

Table S2. The Risk of Bias in Non-Randomized Studies-of Interventions (ROBINS-I) tool.

Reference (first author and year)	Confounding Bias	Selection of Participants	Classification of Interventions	Deviation from Interventions	Missing Data	Measurement of Outcomes	Results Reporting
Byun 2023	Moderate	Moderate	Low	Low	Low	Moderate	Low
Cui 2023	Moderate	Low	Low	Low	Low	Moderate	Low
Elsayed 2022	Moderate	Moderate	Low	Low	Low	Moderate	Low
Jang 2021	Moderate	Moderate	Low	Low	Low	Moderate	Moderate
Jo 2023	CD	Moderate	Low	Low	Low	CD	Low
Kim 2022	Moderate	Moderate	Low	Low	Low	Moderate	Low
Kim 2023	CD	Moderate	Low	CD	Low	Moderate	Low
Lee 2022	CD	Moderate	Low	Low	Low	CD	Low
Muth 2021	Moderate	Moderate	Low	Moderate	Low	Moderate	Moderate
Raina 2022	Moderate	Low	Low	Low	Low	Moderate	Low
Yalcinbayir 2022	Moderate	Moderate	Low	Low	Low	Moderate	Low
Zyablitskaya	Moderate	Moderate	Low	Low	Low	Moderate	Low

Abbreviations: CD, cannot determine.

Table S3. NIH Quality Assessment of Controlled Intervention Studies.

Reference (first author and year)	1	2	3	4	5	6	7	8	9	10	11	12	3	14
Do 2021	N	NA	N	N	N	Y	Y	Y	Y	Y	Y	N	Y	N

Abbreviations: Y, Yes; N, No; CD, cannot determine; NA, not applicable.

Table S4. GRADE Certainty Assessment and Summary of Findings

Author(s): Charles Zhang, Charles Palka, Daniel Zhu, Daniel Lai, Jules Winokur, Treefa Shwani, Margaret M DeAngelis, Andrew Reynolds
Question: Yamane technique compared to sutured-scleral fixation for secondary intraocular lens implantation
Setting: inpatient (hospital), outpatient (ambulatory surgery center)
Bibliography:

Certainty assessment							№ of patients		Effect		Certainty	Importance
№ of studies	Study design	Risk of bias	Inconsistency	Indirectness	Imprecision	Other considerations	Yamane technique	sutured-scleral fixation	Relative (95% CI)	Absolute (95% CI)		
**Final Best-Corrected Visual Acuity (follow-up: mean 5.2 months; Scale from: 0.05 to 1.0)**												
13	non-randomised studies	not serious	serious[a]	not serious	not serious	none	331	406	-	MD **0.01 logMAR lower** (0.06 lower to 0.04 higher)	⊕○○○ Very low	IMPORTANT
**Best-Correct Visual Acuity at 1 month (follow-up: 1 months; Scale from: 0.05 to 1.0)**												
10	non-randomised studies	not serious	not serious	not serious	not serious	none	216	291	-	MD **0.08 logMAR lower** (0.12 lower to 0.03 lower)	⊕⊕○○ Low	CRITICAL
**Surgical Duration**												
6	non-randomised studies	not serious	not serious[b]	not serious	not serious	none	177	234	-	MD **24.68 minutes fewer** (35.9 fewer to 13.46 fewer)	⊕⊕○○ Low	IMPORTANT
**Endothelial Cell Count**												
3	non-randomised studies	not serious	serious[a]	not serious	not serious	none	103	118	-	MD **33.09 endothelial cells fewer** (124.79 fewer to 58.6 more)	⊕○○○ Very low	NOT IMPORTANT
**Secondary Surgical Intervention (follow-up: mean 5.2 months)**												
9	non-randomised studies	not serious	not serious[a]	not serious	not serious	none	14/123 (11.4%)	11/172 (6.4%)	**RR 1.60** (0.57 to 4.51)	**4 more per 100** (from 3 fewer to 22 more)	⊕⊕○○ Low	IMPORTANT
**Cystoid Macular Edema (follow-up: mean 7.1 months)**												
5	non-randomised studies	not serious	serious[a]	not serious	not serious	none	20/256 (7.8%)	30/304 (9.9%)	**RR 0.76** (0.45 to 1.28)	**2 fewer per 100** (from 5 fewer to 3 more)	⊕○○○ Very low	IMPORTANT
**Refractive Error (follow-up: mean 4 months)**												
3	non-randomised studies	not serious	serious[a]	not serious	not serious	none	78	93	-	MD **0.04 diopters lower** (0.33 lower to 0.26 higher)	⊕○○○ Very low	NOT IMPORTANT

CI: confidence interval; **MD:** mean difference; **RR:** risk ratio

Explanations

a. Final follow up times varied across studies from 1 month to more than 12 months
b. The wide range of durations is due to some studies including pars plana vitrectomy in their surgical duration calculation and others not including it. Studies' authors were consistent with reporting surgical duration in that either all cases included PPV or anterior vitrectomy in their calculation of duration or not. Therefore, the difference in surgical duration can be attributed to difference between fixation techniques rather than time spent on vitrectomies.
